# The impact of smartphone addiction on creativity among college students: a moderated mediation model of negative emotion and perceived social support

**DOI:** 10.3389/fpsyt.2025.1567285

**Published:** 2025-05-13

**Authors:** Ju Guo, Xueqian Song, Guiqing Li, Ge Wen

**Affiliations:** School of Management, Chengdu University of Information Technology, Chengdu, China

**Keywords:** smartphone addiction, creativity, negative emotion, perceived social support, college student

## Abstract

**Introduction:**

Smartphone addiction has a negative impact on creativity among college students, which has attracted extensive attention from scholars.

**Methods:**

In this study,we surveyed 2900 Chinese college students using the Smartphone Addiction Scale, Negative Emotion Scale, Perceived Social Support Scale, and Creativity Self-evaluation Scale to explore the impact mechanism of smartphone addiction on creativity.

**Results:**

Results from the hierarchical regression analysis indicated that smartphone addiction has negative influence on creativity in college students (b=-0.158, P<0.001), but positive impacts on negative emotion (b=0.568, P<0.001). The bootstrap analysis indicated that perceived social support not only negatively modulates the impact of negative emotion on creativity, but also modulates the mediating effect of negative emotion on the relationship between smartphone addiction and creativity among college students.

**Discussion:**

These conclusions extend the current research on the hazards of smartphone addiction. According to the results of this study, this article provided practical recommendations for teachers and college students in China.

## Introduction

In the past few years, the development of electronic information technology has driven the advancement of smartphones, making them affordable and easy to use, leading to an exponential growth in global smartphone usage (1). It is expected that the number of smartphones used will reach 7.516 billion by 2026 (2). As of June 2024, China’s Internet penetration rate has run to 78.0%, an increase of 0.5 percentage points from December 2023. Meanwhile, China’s Internet users numbered nearly 1.1 billion (1,099.67 million), up 7.42 million from December 2023, of which the scale of smartphone users has increased to 1.096 billion, with the ratio of Internet users who access the Internet by smartphones was 99.7%, and college students making up the vast majority. The 5th generation mobile communication technology (5G) is characterized by high speed, low latency and large connectivity, making smartphones more powerful. Elhai et al. (3) pointed out that smartphones may improve productivity (e.g., handheld office), promote online interpersonal communication (e.g., social media), expand entertainment (e.g., movies on the Internet), and access information and services anytime, anywhere (e.g., online shopping). The 2024 White Paper on Sleep Health of Chinese Residents, released by the China Sleep Research Association on March 21, 2024, states that 56% of Chinese college students use their smartphones for over eight hours a day. Despite the many benefits provided by smartphones, the overuse of smartphones by college students has led to the emergence of smartphone addiction (SPA), which refers to the psychological or behavioral problems of individuals losing self-control over smartphone use, experiencing difficulties in withdrawal, escaping reality, and inefficiencies in learning, resulting in many adverse impacts on their physical and mental health (4).

Previous studies have confirmed that SPA has physical, psychological and emotional effects on individuals. Jiang and Zhao (5) pointed out that smartphone addicts have severe physical, psychological, and social impairments, and Hu’s (6) study also reached similar conclusions. Elhai et al. (3) pointed that SPA has negative influences on the physical and mental health of college students. Li and Ren (7) believe that SPA in essence is a behavioral addiction that occurs during a person’s interaction with their phone and is a manifestation of unreasonable use of the phone. Lapierre et al. (8) argue that SPA refers to the inability to control and overuse mobile phones, which have adverse effects on an individual’s daily life. Zhang et al. (9) pointed that SPA negatively affects adolescent health. Zeerak et al. (10) insisted that SPA has a negative impact on the academic performance of students. Nambirajan et al. (11) pointed that SPA may lead to sedentary behaviors, potentially impacting physical health adversely. Yang et al. (12) identified a high prevalence of severe SPA, insufficient physical activity, and elevated rates of depressive symptoms among secondary school students.

The multidimensional nature of creativity (CRE) has led to persistent conceptual fragmentation in academic research, with scholars prioritizing distinct analytical frameworks to deconstruct its components (13). The foundational four-P paradigm (Product, Process, Person, Place) systematically categorizes creative phenomena into four interdependent dimensions, requiring investigators to examine how creators’ cognitive capacities, environmental interactions, and operational workflows collectively manifest as socially validated innovations (14). This framework emphasizes that meaningful creativity emerges through synergetic alignment between individual aptitude (e.g., divergent-convergent thinking patterns), contextual catalysts (e.g., collaborative ecosystems), and outcome validation mechanisms ultimately producing artifacts demonstrating contextualized novelty and functional value within specific cultural milieus (15). SPA can have several impacts on the CRE of college students, including structural degradation of cognitive functions (such as atrophy of the prefrontal cortex, imbalance of the dopamine system, and decreased memory encoding efficiency), alienation of thinking patterns (such as shallow information processing inertia, impaired concept jumping ability, and weakened interdisciplinary integration ability), solidification of behavioral patterns (such as immediate feedback dependence, instrumental creativity traps, and fragmented creative patterns), and systematic consumption of psychological resources (such as depletion of attention resources, increased thinking inertia, and inhibition of innovation motivation).

**Figure 1:**
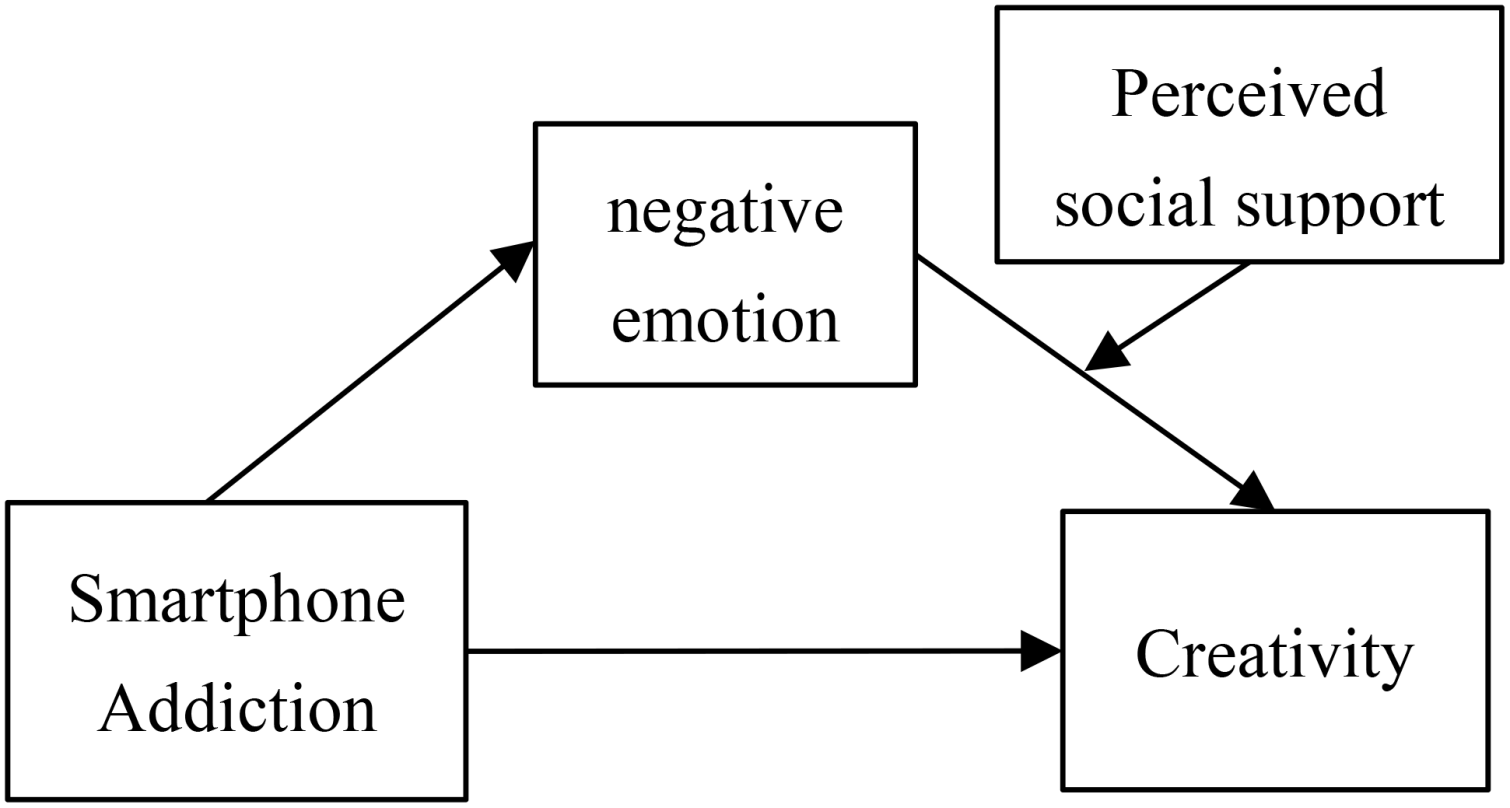
Hypothetical model for the SPA, NEM, PSS, and CRE.

By combing through existing study findings on SPA, we found that previous researches have not paid much attention to the impact mechanism of SPA on college students’ CRE. That is, scholars have yet to fully explain the possible mediating mechanisms as well as moderating effects between SPA and college students’ CRE. Therefore, in order to enrich the research findings on SPA, this study examines the impact of SPA on CRE using a sample of Chinese college students. By introducing negative emotion (NEM) as a mediating variable and perceived social support (PSS) as a moderator variable in the relationship between SPA and CRE, this article constructed a moderated mediation model (**Figure 1**), aiming to investigate whether SPA can increase the level of NEM, and investigate in what manner such increased NEM affects the CRE among college students, and to explore whether NEM mediates the relationship between SPA and CRE, and also to test whether PSS moderates the mediating effect of NEM.

In this model, SPA is seen as the starting point that affects creativity, which can lead individuals to experience NEM such as anxiety, depression, and loneliness while using smartphones. NEM play a mediating role in the model, as they convey the negative impact of SPA on CRE. PSS plays a mediation role, as it can alleviate NEM caused by SPA, thereby reducing the negative impact of these emotions on CRE. In this model, CRE is the dependent variable influenced by SPA, NEM, and PSS. SPA reduces CRE by triggering NEM, while PSS mitigates the negative impact on CRE by alleviating NEM.

## Theory and hypotheses

### The effect of smartphone addiction on college students’ creativity

Although SPA as a new type of addictive behavior has received widespread attention from researchers, there is currently little study on the direct impact of SPA on CRE, and few researches have shown solicitude for the negative impacts of SPA on college students’ CRE. In previous studies, researchers have tested the effect of other addictive behaviors on CRE. For instance, Hajcak’s (16) empirical study found that male college students who participated in two stages of creative experimentation (excessive drinking and non drinking) typically exhibited higher primitive productivity when drunk, but reduced their ability to creatively solve problems, in addition, Holm and Bertolino’s (17) case study on alcohol and drug addiction also yielded similar results, indicating that excessive use of high doses of alcohol and drugs can diminish participants’ CRE.

Chinese researchers have found that individuals with online game addiction behaviors among middle school students exhibit lower creative tendencies, probably because the single mode of information exchange and stereotyped cognitive style of online activities, which excessively stimulate people’s cerebral cortex with a large amount of emotional and fleeting information, changing the original cognitive processing of information, and made individuals no longer willing to actively search for information, but more inclined to passively receive information, which may hinder the development of divergent thinking and thus hinder the performance of creative tendencies among middle school students (18, 19). In addition, some researchers have examined the effects of excessive use of smartphone-related functions on CRE.

In addition to the aforementioned studies on the effects of other addictive behaviors on CRE, recent studies on the effects of SPA on individuals’ cognitive activities have found that SPA has significant effects on cognitive activities related to CRE. For example, Wang et al. (20) examined the differences in inhibitory control among college students with different levels of smartphone dependence using the stop signal paradigm, and found that individuals with high smartphone dependence responded too quickly to non stop signals and too slowly to stop signals, and had poorer inhibitory control than college students with low smartphone dependence. Chen et al.’s (21) study on the effect of overuse of smartphones on inhibitory functioning also had similar results, stating that individuals addicted to smartphones tend to have more conflicts in the early stages of inhibitory function, with obvious inhibitory function defects. As smartphones can be utilized to perform different tasks concurrently, they also make people more susceptible to interference from unrelated stimuli, as they need to abandon the performance of the main task to allow different task information to enter, resulting in impaired inhibitory function of individuals. Hartanto and Yang’s (22) study on SPA and working memory came to similar conclusions, finding that SPA has a negative effect on inhibitory control and working memory, with high addicts exhibiting lower inhibitory and regulatory control.

According to the physiological mechanism of attention and the theory of attention occurrence, SPA can lead individuals to become overly dependent on their phones, spending a lot of attention on social media, online games, and other entertainment applications, resulting in reduced attention to innovation and exploration activities in real life, decreased curiosity and thirst for knowledge about things, and limiting the development of innovation ability. In addition, various messages and social media on smartphones constantly distract college students’ attention, affecting their thinking focus and learning efficiency, and thus affecting their CRE.

In addition, although the popularization of the Internet and smartphones provides a large amount of information and knowledge, it may also lead to the formation of fragmented thinking habits among college students, who are more inclined to obtain information quickly and lack the opportunity for independent and in-depth thinking, making it difficult to form innovative thinking and creative problem-solving ability.

Based on previous empirical studies and theoretical explanations, we made the following research hypotheses:

**Hypothesis 1 (H1):** SPA negatively affects college students’ CRE.

### The mediating effect of negative emotion on the relationship between smartphone addiction and creativity

With the continuous development of mobile Internet technology, which promotes the booming development of smart phones and the use of a large number of APPs, smartphone is no longer the traditional ways of communication, it has become an essential part of college students’ life, and carrying and using cell phone has become a habit of college students. Through the smart phone can not only meet their own food, clothing, housing and transportation and other issues, but also through the smart phone to understand the external information and achieve the purpose of entertainment. However, while smart phones bring convenience to college students, they also have adverse impacts on their physical and mental health.

College students are in the stage of cultivating their values, and their views on various things are not yet mature. At the same time, they are immersed in the online space filled with various information all day long. Various groups of people flaunt their wealth on WeChat, Weibo, short video platforms, and share travel photos, etc., which constantly make college students anxious about comparing themselves with others, resulting in psychological problems such as academic anxiety, family anxiety, appearance anxiety, body anxiety, social anxiety, etc., exacerbating their dissatisfaction with their current learning life and leading to NEM. In addition, the quality of information on the internet varies greatly, with various negative events, fraud incidents, criminal cases, and other information increasing the sense of distrust in college students’ interpersonal relationships, leading to NEM such as social pressure.

Previous researches have also paid attention to the NEM impact of SPA on college students. Zhang et al. (23) reported that reducing the dependence on smartphones among college freshmen can prevent and improve anxiety and depression. Desouky and Abu Zaid (24) pointed out that students with high smartphone dependence spend more time on their phones, lack of communication with teachers and classmates, and can even have family conflicts, which make them more prone to NEM such as anxiety or depression.

Magdalena and Mercedes (25) found that negative emotions also have a predictive effect on smartphone addiction. At the same time, living independently in the university campus, far away from the constraints of parents, makes smartphone addicted college students lack of self-discipline in the management of cell phone use, they spend a lot of time which should be used for learning to passively obtain scattered information from smartphones, such as by constantly brushing short videos, playing online games, brushing the WeChat circle of friends, and other activities that do not require in-depth thought to obtain short-lived happiness and psychological satisfaction, resulting in a disconnect or even a gap between online life and real life, which in turn breeds NEM.

According to the above analysis, we made research hypotheses as follows:

**Hypothesis 2 (H2):** SPA has a positive effect on NEM among college students.

Scholars usually categorize emotions into positive and NEM to explore to explore their impacts on CRE. From the perspective of cognitive mechanisms, positive emotions stimulate CRE and increase creative activity, while NEM inhibit CRE and decrease creative activity. Emotions can affect an individual’s information processing and working memory. Individuals with high working memory can retain more information, which in turn provides more cognitive material for the individual’s associative and thinking processes, increasing the correlation between various cognitive components. Gong and Tang (26) believe that positive emotions can increase cognitive flexibility and improve an individual’s CRE, increasing creative activities. Lu et al. (27) proposed that positive emotions can expand cognitive scope, improve information processing speed, continuously enhance individual CRE, and increase creative activities. Meanwhile, researchers believe that NEM can narrow an individual’s cognitive range, reduce attention, impact CRE, and decrease their creative activities. Du et al. (28) found through experimental research that fear emotions can affect individual CRE and inhibit creative behavior. Liu Xinmei’s (29) study on corporate employees found that positive emotions positively affect CRE, while NEM have a negative impact on employee CRE.

In terms of psychological perspective, emotions can send out different signals to change the individual’s information processing style. Positive emotions release pleasant and relaxing information, making it easier for individuals to initiate information processing to creatively solve problems, while NEM release low and negative information, which increases the individual’s defense mechanism and focuses more on external environmental information, which is not conducive to the development of internal deep thinking and CRE.

When individuals are in a positive emotional state, they can activate a top-down information processing mode and adopt a flexible and inspiring cognitive system, which can stimulate individuals’ cognitive flexibility and improve divergent thinking, thereby enhancing CRE and increasing creative activities. Whereas, individuals in a negative emotional state tend to narrow their thinking and actions within specific ranges, reducing cognitive flexibility and breadth. They use a bottom-up information processing model, which is not conducive to the generation of creative thinking and the expression of CRE, resulting in fewer creative activities.

According to the above explanations, we proposed a hypothesis as follows:

**Hypothesis 3 (H3):** NEM have a negative impact on CRE among college students.

Based on the inference of hypotheses 2 and 3, it can be inferred that SPA may lead to NEM such as anxiety and depression, reducing individuals’ motivation to think and desire to explore, and negatively affecting their innovative thinking and CRE. Therefore, we made a hypothesis as follows:

**Hypothesis 4 (H4):** SPA has a significant negative effect on college students’ CRE through NEM.

### The moderating role of perceived social support on the mediating effect of negative emotion

Social support means to psychological and material supports given by social networking to individuals in order to help them alleviate the stress response when facing stressful events, which is mainly composed of instrumental support (material assistance, operational support, and social contact), psychological support, information assistance, and companion support. Barrera (30) categorized social support into actual and PSS based on its nature differences, and the former refers to the actual behavioral assistance provided by the outside world to individuals, including direct material support and assistance from social networking and group relationships, while the latter refers to the individual’s subjective perception of the level of external support he or she receives from family, friends, co-workers, community organizations, and the government.

According to the social support theory, that is, individuals receive emotional, informational, and material support from the social network, which help to cope with challenges and pressures, enhance psychological resilience, and social adaptability. When college students face the pressure and anxiety caused by SPA, if they can perceive various forms of social support from family and friends, such as emotional encouragement and understanding, material help and support, and informational guidance and advice, these supports can help college students to enhance their self-confidence and to adopt more effective strategies to cope with the pressure and anxiety, so as to decrease the negative effect of NEM on CRE. Meanwhile, when college students perceive a higher degree of social support, they are more inclined to release NEM, alleviate anxiety, stress, and depression, and alleviate the negative effect of NEM on CRE. Combining the elaboration of hypotheses 1 to 4 above, we put forward a hypothesis as follows:

**Hypothesis 5 (H5):** PSS moderates the mediation effect of NEM on the relationship between SPA and CRE.

## Materials and methods

### Participants and data collection

The present study conducted a survey on the college students in China through the distribution of questionnaires on Wenjuanxing, which is an online survey agency. The survey questionnaire takes approximately 5–8 minutes to complete, and each of the participates was compensated with 1 Yuan (RMB), and they can also obtain research results from the author, providing theoretical basis for addressing SPA. Prior to the inquiry, respondents were told of the aim of the investigation and also notified that the data was only for scientific study and would not be used for any other purposes. In order to allay the concerns of answerers and to ensure the quality of data, the research process followed ethical requirements, with respondents answering the questionnaires anonymously and personal information being kept strictly confidential without revealing their personal privacy. We have also set some restrictions to assure the validity of the investigate questionnaire. For example, the questionnaire can only be filled in once by the same IP address to prevent duplication.

This study surveyed college students majoring in management, nursing, computer science and technology, economics, law, and education from provinces such as Sichuan, Yunnan, Guangxi Zhuang Autonomous Region, Zhejiang, Guangdong, and Jiangsu, and collected 2908 data. After removing random and missing ones, 2900 valid questionnaires were received with a validity rate of 99.8%. Among these participants, 1866 (64.32%) are females and 1035 (35.68%) are males, with 63.5% and 36.5% coming from undergraduate schools and specialized (higher vocational) schools, and 53.77% and 46.23% coming from public and private colleges, respectively.

### Measurements

This study adopted the 5-point Likert scale to test the main study variables such as SPA, NEM, and PSS with question items scored on a scale from 1 to 5, indicating strongly disagree to strongly agree. These scales in this article all cite authoritative scales, and some of the item descriptions have been appropriately modified after the pre-survey to fit the research context. The reliability of all scales in this article was measured using Cronbach’s alpha (31).

### Smartphone addiction

We used the scale developed by Leung Louis (32) for diagnosing SPA in adolescents or college students, consisting of 17 items, which were divided into four factors of uncontrollability, withdrawal, avoidance, and inefficacy. We used a Likert 5-point scale, and the higher the score, the higher the level of SPA. In this research, it has a good validity, with a Cronbach’s alpha coefficient of 0.95.

### Negative emotion

We used the short version of the DASS-21 developed by Taouk et al. (33), which consists of 21 items describing the individual’s recent (past week) NEM experiences or corresponding physiological reactions, for example, I feel depressed and frustrated, I feel dry mouth, etc. Participants made judgments about how well the descriptions of the items corresponded to their own situation. A 5-point Likert scale was used, with 1 representing very poor agreement and 5 indicating very good agreement. This scale includes three subscales, such as depression, anxiety, and stress, with each subscale including 7 items. This study has made modifications to some textual expressions to make them more in line with the research context. The alpha coefficient of the scale in this research is 0.97.

### Perceived social support

We used the Perceived Social Support Scale developed by Zimet et al. (34) to test the level of PSS among college students. This questionnaire consists of 12 items, including three dimensions: family support (e.g., I can receive emotional assistance from my family when I need it), friend support (e.g., I can ask my friends for help when I am in trouble), and other assistance (e.g., my leaders, relatives, coworkers, etc., will be there for me when I encounter difficulties). This scale is scored on a 5-point scale (1 = strongly disagree, 5 = strongly agree), and the cumulative scores for all questions represent the individual’s level of PSS, with the higher the score, the better the degree of PSS. In this research, the alpha coefficient of the scale is 0.95.

### Creativity

We adopted the Creativity Self-Evaluation Scale developed by Conner et al. (35) to examine participants’ evaluations of their own CRE. This scale has one item, specifically: please rate your CRE overall on a scale from 0 to 10. Higher scores on the scale indicate that participants perceive themselves to be more creative.

### Data analysis

The data analysis methods used in this article are as follows: First, a Harman’s single-factor test was utilized to check the Common Method Biases (CMB). Second, the Confirmatory Factor Analysis (CFA) was conducted using Amos24.0 to check the discriminant validity between SPA, NEM, PSS, and CRE. Third, descriptive statistical and correlation analysis were conducted using SPSS 24.0. At last, PROCESS Macro (36) was used to verify the moderated mediation model.

## Results

### Reliability analysis

This study used Spss24.0 to check the reliability of scales of SPA, NEM, CRE and PSS, and the results were shown in **Table 1**.

**Table 1:** Reliability analysis of scales.

Measurement	SPA	NEM	PSS
**Cronbach alpha**	0.95	0.97	0.95

χ^²^/df: Ratio of chi square to degrees of freedom.

RMSEA, Root Mean Square Error of Approximation; GFI, Goodness-of-Fit Index; CFI, Confirmatory Fit Index; NFI, Normed Fit Index; NNFI, Non-Normed Fit Index.

### Common method biases

Since all variables of this measurement were reported by the participants, in order to avoid the influence of CMB on the results, Harman’s single-factor test (37) was used, and the percentage of variance explained by the first factor to the total variance explained was used as the judgment basis. The results showed that the variance explained rate of the first factor was 29.84%, which did not exceed the critical criterion of 40%, suggesting that there was no significant common method bias and further analysis could be conducted.

### Confirmatory factor analysis

We adopted Amos24.0 to conduct CFA on the 4-factor model composed of SPA, NEM, PSS, and CRE, and the results were compared with single-factor and 3-factor models, and it was found that the fitting degree of the 4-factor model, which with x^2^/df=1.98, RMSEA=0.07, GFI=0.94, NNFI=0.97, NFI=0.95, and CFI=0.99, had significant advantages over other models. Therefore, the discriminant validity of the 4-factor model was higher than other ones (**Table 2**).

**Table 2:** Results of confirmatory factor analysis.

Model	$x^{2}/df$	RMSEA	GFI	CFI	NFI	NNFI
Single-factor	36.16	0.26	0.53	0.46	0.19	0.45
3-factor^a^	24.73	0.18	0.51	0.78	0.62	0.79
3-factor^b^	26.47	0.17	0.45	0.76	0.51	0.85
3-factor^c^	25.32	0.15	0.46	0.87	0.53	0.83
4-factor	1.98	0.07	0.94	0.99	0.95	0.97

n = 2900; the one-factor model considers all variables as one factor. The 3-factor model (a) takes PSS and CRE as 2 separate factors, while considering SPA and NEM as one factor. The 3-factor model (b) takes SPA and CRE as 2 separate factors, while considering NEM and PSS into one factor. The 3-factor model (c) treats SPA and NEM as 2 separate factors, while considering PSS and CRE into one factor. The 4-factor model treats 4 variables as separate factors.

### Descriptive statistics and correlation analysis among study variables

**Table 3** presented the results of descriptive statistics and correlation analysis among the main research variables, which indicated positive correlations between SPA and NEM (r= 0.564, p< 0.01), and PSS (r=0.139, p< 0.01), while a negative correlation between SPA and CRE (r=-0.158, p< 0.01). Meanwhile, NEM has a negative correlation with PSS (r=-0.089, p< 0.01), also a negative correlation with CRE(r=-0.158, p< 0.01), and PSS has a positive correlation with CRE (r=0.117, p< 0.01). Therefore, correlations between the main variables, namely SPA, NEM, PSS, and CRE, were statistically significant and basically in accordance with our research hypothesis, thus, further hierarchical regression analysis (HRA) can be conducted.

**Table 3:** Descriptive statistics and correlations of all variables.

Variable	Mean	Standard Deviation	1	2	3	4	5	6	7	8
1 Gender	1.640	0.479	–							
2 Grade	1.690	0.861	0.060**	–						
3 School level	1.370	0.482	-0.102**	0.111**	–					
4 School nature	1.460	0.499	-0.246**	0.058**	0.659**	–				
5 SPA	2.984	0.749	0.039*	0.087**	-0.054**	-0.040*	–			
6 NEM	2.353	0.872	-0.033	0.071**	0.055**	0.038*	0.564**	–		
7 PSS	3.655	0.789	0.129**	-0.038*	-0.055**	-0.035	0.139**	-0.089**	–	
8 CRE	7.123	1.910	-0.112**	0.038*	0.050**	0.065**	-0.158**	-0.207**	0.117**	–

Gender: 1 = male, 2 = female; Grade: 1 = Freshman, 2 =Sophomore, 3 = Junior, 4 = Senior; School level: 1 = Undergraduate colleges, 2 = Specialized (vocational) colleges; School nature: 1 = Public universities, 2 = Private universities. SPA, SPA; NEM, negative emotion; PSS, perceived social support; CRE, creativity. n = 2900, * p < 0.05 (double tails), ** p < 0.01 (double tails).

### Mediating effect analysis

To check the effect of SPA on CRE and the mediated role of NEM, we used SPSS 24.0 to conduct Hierarchical Regression Analysis (HRA) on the data, and results can be seen in **Table 4**. Model 2 is a regression model of the dependent variable (NEM) on the independent variable (SPA), and Model 4-Model 5 are regression models of the dependent variable (CRE) on the independent variable (SPA). As presented in **Table 4**, SPA has a significant negative effect on CRE in M4 ( β=−0.158,p<0.001 ). Thus, hypothesis1 was verified.

**Table 4:** The results of hierarchical regression.

Variable type	NEM		CRE			
	M1	M2	M3	M4	M5	M6
Control Variables						
Gender	-0.034	-0.05	-0.107	-0.103	-0.112	-0.13
Grade	0.068	0.015	0.041	0.055	0.058	0.063
School level	0.047	0.083	0.018	0.008	0.024	0.027
School nature	-0.006	-0.007	0.024	0.024	0.023	0.015
Independent Variable						
SPA		0.568***		-0.158***	-0.051*	-0.105***
Mediating Variable						
**NEM**					-0.188***	-0.485***
Interaction						
NEM×PSS						0.355***
**R^2^**	0.08	0.327	0.016	0.041	0.064	0.099
**F-value**	6.06***	281.713***	11.794***	24.51***	33.154***	45.20***
**△R^2^**	0.008	0.319	0.016	0.025	0.024	0.34
**△F**	6.06***	1372.838***	11.794***	74.182***	73.308***	109.983***

n = 2900; ***p < 0.001, **p < 0.01, *p < 0.05. The dependent variable of M1-M2 is NEM (negative emotion), and the dependent variable of M3-M5 is CRE (creativity).

With the addition of the mediating variable (NEM), SPA has an obvious positive impact on NEM (M2: β=0.568,p<0.001 ), and at the same time, NEM has an obvious negative effect on CRE (M5: β=−0.188,p<0.001 ), therefore, hypotheses 2 and 3 were verified. **Table 4** shows that the direct effect of SPA on CRE is weakened from 0.158 (M4: β=−0.158,p<0.001 ) to 0.051(M5: β=−0.051,p<0.05 ) due to the addition of the mediating variable, which indicates that SPA has both a direct effect on CRE and an indirect impact through the mediating role of NEM, that is, NEM has a partial mediating effect on the relationship between SPA and CRE. In addition, the bootstrap test indicated that this mediation effect is significant with a mediating effect value of 0.247, 95% CI = [-0.315, -0.180] and excluding 0 (**Table 5**), and that the mediation effect is 61.14% of the total effect. Thus, hypothesis 4 was verified.

**Table 5:** Analysis of mediating effect of NEM.

Effect type	b	Boot SE	Bootstrap 95% CI	Efficiency Ratio
Total Effect	-0.404	0.047	[-0.495, -0.312]	
Direct Effect	-0.157	0.056	[-0.267, -0.047]	38.86%
Indirect Effect	-0.247	0.035	[-0.315, -0.180]	61.14%

Model 4 in the PROCESS macro. Bootstrap samples = 5,000. b is a nonstandard regression coefficient, SE is a Std Error, and CI is a confidence interval.

### Moderated mediation effect analysis

After standardizing scores of SPA, NEM, PSS, and CRE, a interaction term was calculated, which was NEM×PSS. HRA was done after controlling gender, grade, school level and school nature factors (**Table 4**), indicating that the interaction term between NEM and PSS has a significant effect on CRE (M6: β=0.355,p<0.001 ).

**Figure 2:**
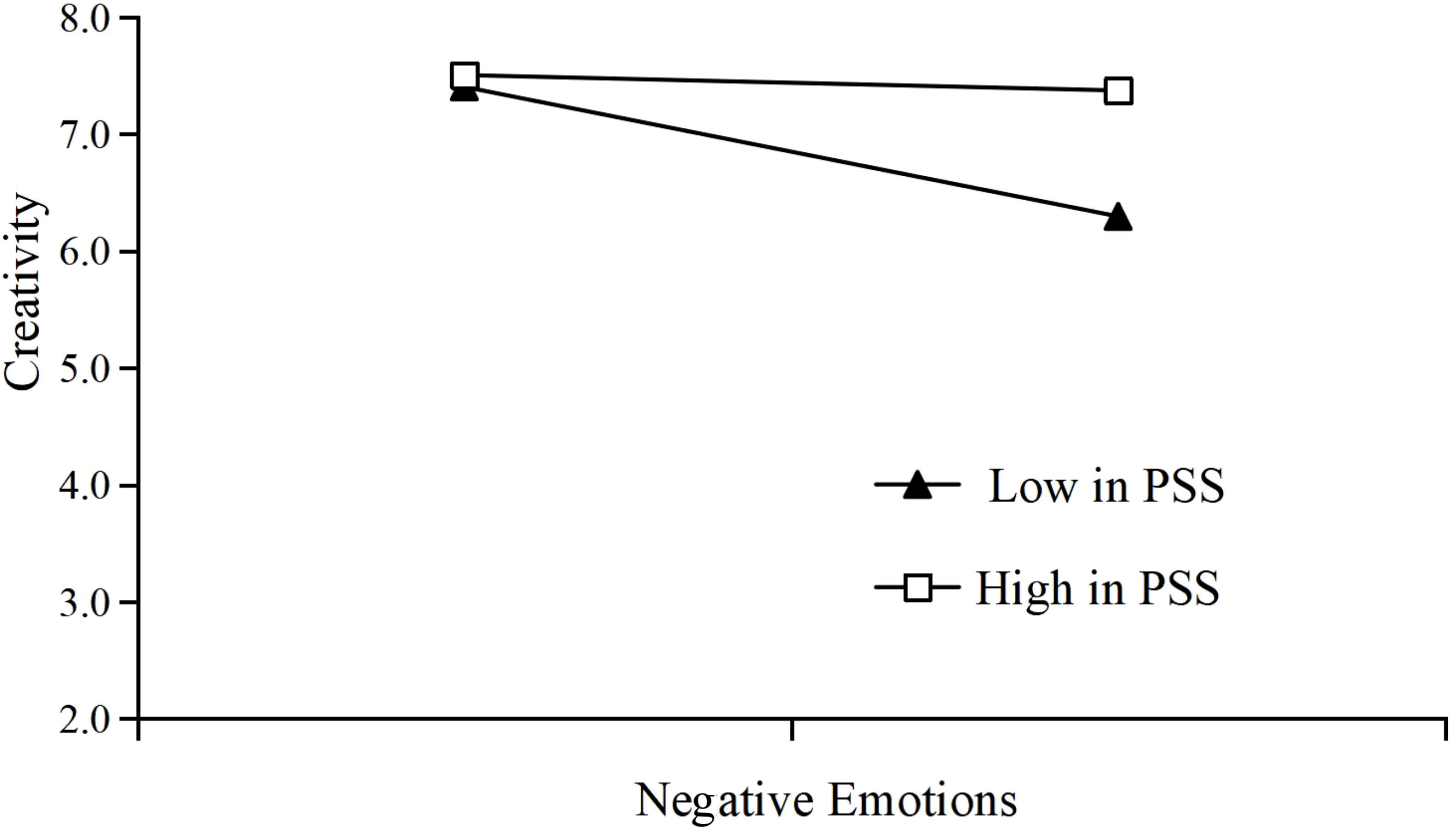
The moderating effect of perceived social support.

In order to further analyze the moderating effect of PSS on the relationship between NEM and CRE, this study divided PSS into high-level and low-level groups based on the standard deviation principle, and conducted a simple slope test (**Figure 2**), which combined with the results in **Table 6**, shows that for college students with high levels of PSS, the negative effect of NEM on CRE was not significant (b = -0.076, 95% CI = [-0.184, 0.033], ns), while for those with low levels of PSS, the negative effect of NEM on CRE was significant ( b=-0.635, 95% CI=[-0.754, - 0.517],p<0.001 ), indicating that the negative impact of NEM on CRE is negatively moderated by PSS. In other words, the higher the level of PSS among college students, the less significant the impact of NEM on CRE.

**Table 6:** The conditional effects of NEM on CRE at different levels of PSS.

Mediator	Condition	Effect	SE	p	LLCI	ULCI
PSS	Low PSS	-0.635	0.06	0.000	-0.754	-0.517
	Middle PSS	-0.356	0.048	0.000	-0.451	-0.261
	High PSS	-0.076	0.055	0.171	-0.184	0.033

We used model 14 of Hayes’ SPSS PROCESS macro (36) to check the indirect effects of NEM at different levels of PSS. The results can be seen in **Table 7**, indicating the indirect effect of NEM on the relationship between SPA and CRE is significant when the level of PSS is low ( b=−0.417,SE=0.047,95%CI=[−0.509,−0.324] ) or medium ( b = -0.233,  SE=0.035,95% CI = [-0.301, - 0.163] ), but not significant when PSS is high (b= -0.05, SE= 0.044, 95% CI= [-0.137, 0.036]). In addition, based on the Hayes (38) method to check the moderated mediation effect, the index of moderated mediation is 0.233, and the Bootstrap 95% CI is  [0.161, 0.302] , excluding zero. In general, PSS moderated the mediating effect of NEM on the relationship between SPA and CRE. In other words, the moderated mediation analysis shown that the lower the PSS, the stronger the mediation role of NEM in the relationship between SPA and CRE. Thus, hypothesis 5 was validated.

**Table 7:** Analysis of moderated mediation.

Mediator	Indirect Effects					Index of moderated mediation			
	Condition	Effect	BootSE	Boot LLCI	Boot ULCI	Index	BootSE	Boot LLCI	Boot ULCI
PSS	Low PSS	-0.417	0.047	-0.509	-0.324	0.233	0.036	0.161	0.302
	Middle PSS	-0.233	0.035	-0.301	-0.163				
	High PSS	-0.050	0.044	-0.137	0.036				

Model 14 in the PROCESS macro. Bootstrap samples = 5,000. The PSS is conditioned on the mean plus or minus one standard deviation. Both NEM and PSS were centralized prior to analysis.

## Discussion

### Main findings

This study explored the mechanism by which SPA affects the CRE of college students in China, with the moderated mediation model of NEM and PSS, making the following important findings. First, based on the results of HRA (**Table 4**), we found that SPA has an obvious adverse effect on CRE among college students (M4: β=−0.158,p<0.001 ), which is consistent with previous research findings, such as scholars Wang et al. (20), Chen et al.’s (21), and Hartanto and Yang’s (22), who have explained from different perspectives that SPA has an obvious adverse effect on CRE among college students. Second, we found that NEM partially mediates the relationship between SPA and CRE, as the negative effect of SPA on CRE weakens from -0.158 (M4: β=−0.158,p<0.001 ) to -0.051 (M5: β=−0.051,p<0.05 ) when the mediator variable (NEM) was added into the regression model, and the mediation effect is 61.14% of the total effect. Third, the combined results of the HRA (**Table 4**), conditional effects analyses of NEM on CRE at different levels of PSS (**Table 6**), and simple slope analyses (**Figure 2**) indicated that PSS negatively moderates the negative impact of NEM on CRE, as the coefficient sign of the interaction term between NEM and CRE is opposite to that of the regression coefficient of CRE on NEM, in other words, the weaker the effect of NEM on CRE is for college students with a higher level of PSS. Finally, results of moderated mediation analysis (**Table 7**) indicated that the indirect impact of SPA on CRE through NEM is negatively moderated by PSS, suggesting that the higher the level of PSS among college students, the weaker the effect of SPA on CRE through NEM.

### Theoretical implications

First, this research checked the moderated mediation model of NEM and PSS, to analyze the mechanism of SPA on CRE. Specifically, building on the theoretical framework of Tittytainment and Social Support Theory, we constructed the moderated mediation model, founding that SPA negatively affects CRE through NEM, and the mediating effect of NEM is negatively moderated by PSS. These research findings extended previous study results.

Second, previous studies have focused more on other types of addiction, such as alcohol addiction (39–41), drug addiction (42, 43), food addiction (44, 45), behavioral addiction (46, 47), etc., and less on SPA, as well as the negative impact of SPA on CRE among college students. This research suggest a new mechanism to explain the negative effect of SPA on the CRE of college student, that is, SPA has a negative effect on the CRE of college students through the partial mediation effect of NEM, expanding existing research findings.

Finally, based on the conclusion of this article, it can be concluded that SPA has caused many negative impacts on college students, and it is very necessary that school teachers, parents and college students themselves should pay great attention to the negative hardness brought by SPA, and should take positive countermeasures. The findings of this paper can provide theoretical bases for eliminating SPA.

### Practical implications

This study proposes the following practical implications from the perspectives of schooling and college students’ self-regulation. According to the Field Habitus Theory, the objective field and subjective habituation are inseparable, the field is the field of habit, and the habit that is separated from the field does not exist. We regard universities as fields and college students’ SPA as habitus, and from the perspective of school education, we propose the following suggestions for cultivating creative thinking and CRE in students’ use of smartphones:

Firstly, limiting usage time is one of the important means to cultivate students’ creative thinking and CRE. Prolonged smartphone use may lead to students becoming too addicted and unable to concentrate on creative thinking. Therefore, reasonable time limits for smartphone usage should be established to ensure that students have enough time to engage in other more beneficial activities. This can be done by setting up a smartphone use schedule, setting time slots for smartphone use, and prohibiting smartphone use during specific time periods.

Secondly, encouraging diversified use of smartphones is also an important way to cultivate students’ creative thinking and CRE. In addition to using smartphones for entertainment and social activities, students can also be guided to learn and train their creative thinking through smartphones. Schools should develop some beneficial mobile applications, such as mind maps, scientific experiment simulations, etc., to help students take the initiative in creative thinking activities. Teachers should also actively guide students to use their smartphones creatively, such as recording beautiful things by taking photos and recording their creative ideas with smartphones.

Finally, engaging in creative thinking activities is an important way to cultivate students’ CRE. Schools can organize some creative thinking activities, such as art exhibitions, creative writing competitions, to encourage students to show and share their creative achievements. In addition, student creative teams can be formed to carry out innovative projects to enhance students’ CRE.

From the perspective of self-restraint of college students, we put forward the following suggestions: Firstly, to overcome SPA, college students need to enhance their self-control and awareness, better resist the temptation of smartphones, and maintain a positive emotional state. At the same time, cultivate one’s ability of delayed gratification, stay away from the short-lived pleasure brought by low-grade fun, and focus on cultivating deep thinking and CRE. Secondly, if college students’ dependence on smartphones seriously affects their normal learning and life, they can seek social support. Family and friends can provide emotional support and advice to help college students get rid of SPA. Finally, college students should maintain a positive and optimistic mindset, learn to adjust their emotions and stress, maintain physical and mental health, resist the temptation of smartphones, and avoid NEM caused by SPA. Finally, college students should maintain a positive and optimistic attitude, learn to adjust their emotions and stress, and maintain physical and mental health, as well as learn to resist the temptation of smartphones to avoid NEM caused by SPA.

### Limitations and outlook

The limitations of this study should be mentioned. First, only Chinese school students were surveyed, investigating the effects of SPA on CRE among college students from different countries would more accurately reveal the negative effects of SPA on college students’ CRE. Secondly, this study mainly used self-report method, using reports from peers, parents, or teachers to assess SPA would contribute to the validity of the study. Finally, there may be other mediating and moderating variables, such as sense of meaning in life and psychological resilience that may also play a mediating or moderating role in the relationship between SPA and CRE among college students.

## Conclusion

This research indicated that SPA is negatively related to CRE among college students, and positively related to NEM, and NEM has a partial mediation effect on the relationship between SPA and CRE, while the PSS moderates the second part of the mediation process. For college students with high level of PSS, SPA only has a direct effect on CRE, however, for those with low level of PSS, there is an indirect effect mediated by NEM alongside a direct effect. This moderated mediation model combines some perspectives of the Tittytainment, Social Support Theory, and Field Habitus Theory, enriching studies of the impact mechanism of SPA on college students’ CRE.

## Data Availability

The raw data supporting the conclusions of this article will be made available by the authors, without undue reservation.
